# The VirB System Plays a Crucial Role in *Brucella* Intracellular Infection

**DOI:** 10.3390/ijms222413637

**Published:** 2021-12-20

**Authors:** Xue Xiong, Bowen Li, Zhixiong Zhou, Guojing Gu, Mengjuan Li, Jun Liu, Hanwei Jiao

**Affiliations:** 1 College of Veterinary Medicine, Southwest University, Chongqing 402460, China; xx136981@email.swu.edu.cn (X.X.); libowenswu@email.swu.edu.cn (B.L.); zzx449090405@email.swu.edu.cn (Z.Z.); ggj506339263@163.com (G.G.); lkklmj1997@swu.edu.cn (M.L.); 2 Changchun Veterinary Research Institute, Chinese Academy of Agricultural Sciences, Yujinxiang Street 573, Changchun 130122, China; 3 National Center of Technology Innovation for Pigs, Chongqing 402460, China; 4 Veterinary Scientific Engineering Research Center, Chongqing 402460, China; 5 Immunology Research Center, Medical Research Institute, Southwest University, Chongqing 402460, China

**Keywords:** *Brucella*, VirB system, type IV secretion, effector, intracellular infection

## Abstract

Brucellosis is a highly prevalent zoonotic disease caused by *Brucella*. *Brucella* spp. are gram-negative facultative intracellular parasitic bacteria. Its intracellular survival and replication depend on a functional virB system, an operon encoded by VirB1–VirB12. Type IV secretion system (T4SS) encoded by the virB operon is an important virulence factor of *Brucella*. It can subvert cellular pathway and induce host immune response by secreting effectors, which promotes *Brucella* replication in host cells and induce persistent infection. Therefore, this paper summarizes the function and significance of the VirB system, focusing on the structure of the VirB system where VirB T4SS mediates biogenesis of the endoplasmic reticulum (ER)-derived replicative *Brucella-*containing vacuole (rBCV), the effectors of T4SS and the cellular pathways it subverts, which will help better understand the pathogenic mechanism of *Brucella* and provide new ideas for clinical vaccine research and development.

## 1. Introduction

Brucellosis is a debilitating zoonotic disease affecting animals and humans all over the world [1]. The sick animals are characterized by infertility, abortion and decline in milk production, resulting in great economic losses to the livestock husbandry industry [2]. Human disease is caused by direct contact with the blood or tissue of infected animals or by consumption of contaminated dairy products, with clinical symptoms such as intermittent fever, arthritis, orchitis, hepatitis, and depression [2,3]. *Brucella* species frequently isolated from job-related infections are *Brucella melitensis*, *Brucella abortus*, *Brucella suis* and *Brucella canis* [1]. *Brucella* spp. are gram-negative; their main target cells are macrophages, dendritic cells and trophoblast cells [4], and the main virulence factors are lipopolysaccharides (LPS), T4SS and the BvrR/BvrS system [5].

*Brucella* causes host pathogenesis through survival and replication in target cells, an intracellular process dependent on T4SS encoded by the VirB operon [6]. During intracellular infection, VirB T4SS is rapidly activated and reaches its maximum activity in five hours. When the replication niche is established, VirB T4SS is inhibited. During this period, VirB T4SS can subvert the cell pathway of host cells, which is beneficial to the bacterial reproduction and pathogenicity [7]. Although the mechanism of the *Brucella* VirB system in the intracellular circulation has been largely elucidated, there are still some deficiencies. Therefore, this paper will elaborate on the mechanism of VirB action to provide a theoretical basis for the pathogenesis of *Brucella* and clinical vaccine development.

## 2. Composition of the *Brucella* VirB Operon

The VirB system was first discovered in *Brucella suis*, subsequently confirmed to be present in all *Brucella* species and highly conserved [8]. It is composed of 12 open reading frames (ORFs) co-linked and located on the SpeI fragment of chromosome 2 [9,10]. In the VirB region of *Brucella*, an operon is formed by VirB1–VirB12, whose expression is regulated by environmental signals [11]. Hartigh et al. [12] carried out infection experiments in mice with the nonpolar VirB1 mutant (Δ *virB1*) and nonpolar VirB2 mutant (Δ *virB2*). Facts have proved that VirB2 plays an important role in the continuous infection of mice caused by *Brucella*, but VirB1 is dispensable [12]. Similarly, although VirB12 is able to encode a 17 kDa protein that elicits a host immunogenic response during infection, it is not essential for persistent infection in mice and macrophages [13]. In addition, VirB3–VirB11, except for VirB7, are important components of *Brucella* virulence in mice [14] (Table 1).

**Figure 1:**
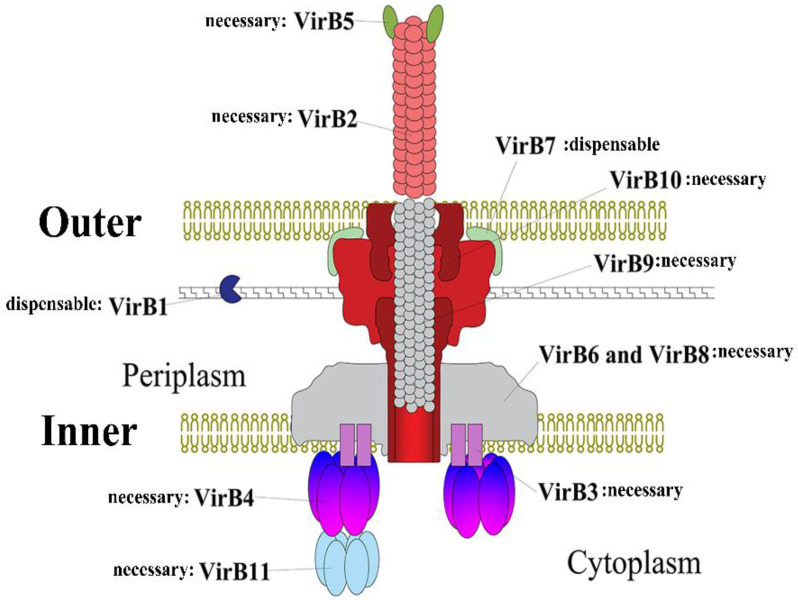
VirB system structure. Prototype of the *Brucella* VirB system, *Agrobacterium tumefaciens* VirB/D4 system. The VirB system of *Brucella* is composed of VirB1-VirB12 and is assembled into three interconnected compartments: the inner membrane complex (VirB3, VirB4, VirB6 and VirB8), the outer membrane core complex (VirB7, VirB9 and VirB10) and the external pilus (VirB2 and VirB5). Two ATPases (VirB4, VirB11) provide the energy for VirB T4SS assembly and substrate transfer. Presently, the exact location of VirB11 relative to VirB4 is not clear, whether it can accumulate under VirB4 or on one side of the cytoplasm.

Each gene in the *Brucella* VirB operon has a specific function and encodes proteins with different structures and functions. VirB proteins can be divided into four groups according to their functions: ATPase, core components, surface exposure components and other components (Figure 1).

### 2.1. ATPase

VirB4 and VirB11 are ATPases composed of two domains (NTD and CTD). VirB4 usually appears as a monomer, dimer or hexamer which provides energy for T4SS assembly and substrate transport through NTP binding [15,16]. The active function of ATP is performed by a six-ring compound formed by the interaction between monomers [17]. Phospholipids can significantly enhance the active function of ATP [17,18].

### 2.2. Core Component VirB6–VirB10

The core components consist of VirB6–VirB10, which are present in the periplasm and contain distinct domains [19] (Figure 1). They interact with each other to form a translocation channel for T4SS, thereby transporting effectors into the host cell [19]. Among them, VirB6 is an endosomal protein with a periplasmic n-terminal, five transmembrane domains and a cytoplasmic c-terminal, and the n-terminal periplasmic domain can interact with VirB8 and VirB10, which is essential for secreting substrates through the inner membrane [19,20,21]. Bound to VirB6, VirB8 exists as a dimer, containing a periplasmic domain, a single transmembrane helix structure and a cytoplasmic c-terminus [22]. The amino acid residues at the dimerization interface determine the function of VirB8 [22,23]. VirB10 can pass through the entire outer membrane of *Brucella* T4SS structure. Therefore, VirB10 is an important functional protein for *Brucella* [21]. The protein consists of an N-terminal domain; it is found that the domain contains four parts: a cytoplasmic component, a TM helix, a bendable component and a spherical CTD [16,24]. These four parts lead to the central function of the VirB10 protein in T4SS, that is, connecting many different proteins and sending interactions so as to mediate the signal transmission process [24]. VirB9 encapsulated by VirB10 (Figure 1) is a periplasmic protein with two domains of NTD and CTD; can interact with VirB7 to form the inner wall of T4SS core complex [16,25]. While VirB7 is a lipoprotein whose n-terminus is acetylated and inserted inside the outer membrane, the remainder of the lipoprotein is located in the periplasm, and the lipidation is essential for maintaining the stability of T4SS [16,26].

### 2.3. Surface-Exposed Components VirB2 and VirB5

The surface-exposed component contains VirB2 and VirB5 (Figure 1), which interact to form T-pilus [16]. VirB2 is the main component of T-pilus, forms a columnar structure on the bacterial surface to transfer the effector protein and also delivers the target signal peptide via the inner membrane [16,27]. VirB5 located at the tip of T-pilus is a minor component of T-pilus that targets host cell receptors as a specific adhesin and helps to integrate the major component VirB2 into T-pilus [28,29].

### 2.4. Other Components VirB1 and VirB12

Other components include VirB1 and VirB12. VirB1 does not bind to any other VirB protein and exist alone in the periplasm; it is the first product of the VirB operon; can cleave the β-1,4-glycosidic bond between MurNAc (N-acetylmuramic acid) and GlcNAc (N-acetylglucosamine) [24]. Its n-terminus is a lytic transglycosylase, which may facilitate T4SS assembly by local destruction of peptidoglycans [15]. The c-terminal protein VirB1* can be cleaved from VirB1 and secreted outside bacterial cells, interacting with the T-pilus subunit to promote T-pilus assembly [30]. The exact location of VirB12 is still unclear; it is only confirmed that it is a cell surface protein that induces antibody response during animal infection [31,32].

## 3. *Brucella* Type IV Secretion System

### 3.1. Formation of T4SS

T4SS is encoded by the VirB operon and divided into three basic parts: the inner membrane complex (IMC: VirB3, VirB4, VirB6, VirB8), the outer membrane complex (OMC: VirB7, VirB9, VirB10) and the outer column (VirB2, VirB5) [16]. OMC is a large structure inserted into the inner membrane and similar to the core complex. It is connected by a flexible central stalk to a larger complex, IMC [24]. Among all the VirB proteins, VirB4 and VirB11 have ATPase activity; they are located in the cytoplasm, providing energy for T4SS assembly or substrate transport [20]. VirB4 can interact with itself, VirB8, VirB10 and VirB11; its stability depends on the formation of the VirB7–VirB9 heterodimer, while VirB6 also affects the stability of VirB4. Besides VirB4, VirB11 can also interact with other proteins to form the VirB11–VirB9–VirB7–VirB2/VirB5 action pathway [15]. In the basic structure of T4SS, the substrate transmembrane transport channel in the periplasm connects the outer membrane and the inner membrane. The inner membrane of the channel is composed of VirB3, VirB6 and VirB8 [20,21,33]. VirB8 acts as an assembly factor to bring VirB9 and VirB10 to the poles of bacteria; then, the intermediate complex VirB9 interacts with VirB10 to induce conformational changes and reduce the level of nonproductive aggregation, and then VirB9 dissociates the VirB8−VirB10 dimer, separates VirB10 from VirB8 [34]. During T-pilus (the outer column) biogenesis, VirB2 is first absorbed by the inner membrane; subsequently, VirB5 interacts with VirB8 and VirB10 with the assistance of the ATPases VirB4 and VirB11, which may be necessary for VirB5 binding to VirB2, and is then processed and assembled to form T-pilus [32]. In non-substrate-contacting VirB proteins, VirB4 coordinates substrate translocation to the VirB6 and VirB8 subunits; thereafter, VirB3, VirB5 and VirB10 facilitate substrate translocation from VirB6 and VirB8 to the VirB2 and VirB9 subunits [35]. A portion of VirB proteins interact with each other to maintain T4SS stability. The VirB4–VirB8–VirB5–VirB2 interaction promotes the assembly and stability of T4SS, the binding of VirB1 with VirB8 and of VirB9 with VirB11 is also an important factor in promoting T4SS transmembrane assembly [36,37]. In conclusion, formation of T4SS depends on the interaction of various VirB proteins, but the mechanism of this interaction is not sufficiently clear, which needs to be further studied.

### 3.2. T4SS Effectors

Although T4SS was discovered in *Brucella* spp. about 15 years ago, the T4SS effectors were confirmed only recently. A candidate protein can be identified as a T4SS effector protein if it meets two criteria. First, the protein must be secreted by VirB T4SS. Second, this protein must be able to enter the host cell [7,8]. The first criterion can be verified by constructing a T4SS-defective strain; the second standard can be verified by TEM-1 lactamase or calmodulin-dependent adenylate cyclase assays [8]. There are currently 15 known VirB T4SS effectors: RicA, VceC, VecA, BtpA, BtpB, BspA, BspB, BspF, BPE005, BPE123, BPE043, BPE275, SepA, BspC, and BspE. They manipulate host cell pathways and responses [7,8].

#### 3.2.1. RicA

RicA is a single-domain protein composed of 175 amino acids assembled in the form of a trimer [38]. Each monomer consists of a left-handed parallel β-helix and an α-helix that is inversely parallel to the β-helix, with 13 hydrogen bonds formed at the interface where each two monomers interact. The three-dimensional structure of RicA can be divided into two parts: the n-terminal LβH and the c-terminal α-helix. LβH is a protein superfamily including acetyltransferases, ferripyochelin-binding proteins, acyltransferases, carbonic anhydrases and some proteins of unknown functions [39]. In LβH, there are three β-layers, two of which are embedded in the trimeric structure and the other is externally exposed [38,39,40]. Rab2 GTPase is a cellular host factor critical for *Brucella* intracellular proliferation. RicA can interact with it in two possible interfaces; one is a β-lamellar interface and the other is a ring called the isoleucine–glycine–phenylalanine–proline (IGFP) loop [39]; RicA relies on its molar affinity to bind to guanosine diphosphate (GDP) and non-GDP forms of the Rab2 GTPase [40].

#### 3.2.2. VceC and VecA

VceC is highly conserved in all sequenced *Brucella* genomes, contains 418 amino acids; the 20 amino acids at the c-terminal are necessary for its transfer to the host cell after entering the host cell; it is activated by a quorum-sensing regulatory protein VjbR and targets the endoplasmic reticulum. The interaction with the endoplasmic reticulum requires 37 amino acids at the n-terminal of the transmembrane domain [41,42,43].

VceA contains 105 amino acids, is identical to VceC, conserved in all sequenced *Brucella* genomes, shares an 18-base-pair (bp)-long conserved promoter with VceC; transcription of VceA is also regulated by VjbR [8,44]. VecA is related to autophagy and apoptosis. The expression levels of the autophagy genes of Atg5 and LC3-II in the VceA mutants constructed by Zhang et al. were higher than those in the wild type, while the expression levels of the autophagy genes of p62 and LC3-I were decreased. Atg5 and LC3-II are genes that promote autophagy. P62 and LC3-I are genes that inhibit autophagy, indicating that VceA has a function of inhibiting autophagy. At the same time, the content of caspase-3 mRNA in VceA mutants decreased and the expression level of Bcl-2 mRNA increased. Caspase-3 is a gene that promotes apoptosis, Bcl-2 is an anti-apoptosis gene, indicating that VceA can promote apoptosis [43].

In conclusion, VceC and VceA are important T4SS effectors which have important effects on autophagy and apoptosis.

#### 3.2.3. BtpA and BtpB

The BtpA gene is located in a 20 kb genomic island on chromosome 1, present in *Brucella melitensis*, *Brucella abortus* and absent in *Brucella* *suis*. BtpA is a protein composed of 250 amino acids that contains two domains: one is the n-terminal domain which can bind to phosphoinositide and the other is a c-terminal TIR domain [45,46,47,48]. In addition to subverting the innate immune signaling pathway, purified BtpA has also been shown to inhibit CD8+ T cell-mediated killing response, suggesting it may also control the acquired immune response [47].

Unlike BtpA, BtpB, which is present in all *Brucella* species, is formed by 292 amino acids and can downregulate the total level of NAD in host cells through direct enzymolysis of metabolic cofactors, thus directly controlling host energy metabolism [49,50].

BtpA and BtpB play different roles in host cells. BtpA mainly causes host immunity, BtpB is mainly related to energy metabolism.

#### 3.2.4. BspA, BspB, BspF

BSPA, BspB and BspF consist of 191, 187 and 428 amino acids, respectively, and contain the DUF2062 domain (unknown functional domain 2062, Pfam database), the SCOP domain (flanked by two transmembrane domains) and the Gcn5-associated N-acetyltransferase (GNAT) family acetyltransferase domain [51].

#### 3.2.5. Other Effectors

T4SS effectors also include BPE005, BPE123, BPE043, BPE275, SepA, BspC and BspE. BPE005, BPE275 and BPE043 are widespread in a variety of bacteria, while BPE123 is only found in *Bartonella bacilliformis*, *Ochrobactrum anthropi* and *O. intermedium* [8]. In *Brucella*, BPE123 can act on the α-enolase of host cells to promote the intracellular life of *Brucella* [52]. At the same time, SepA is involved in the early stage of intracellular survival; its secretion occurs 30 min after infection [53]. There is a lack of studies on the structure and function of BPE043, BPE275, BspC, and BspE.

## 4. Effect of VirB T4SS on the Intracellular Circulation of *Brucella*

**Figure 2:**
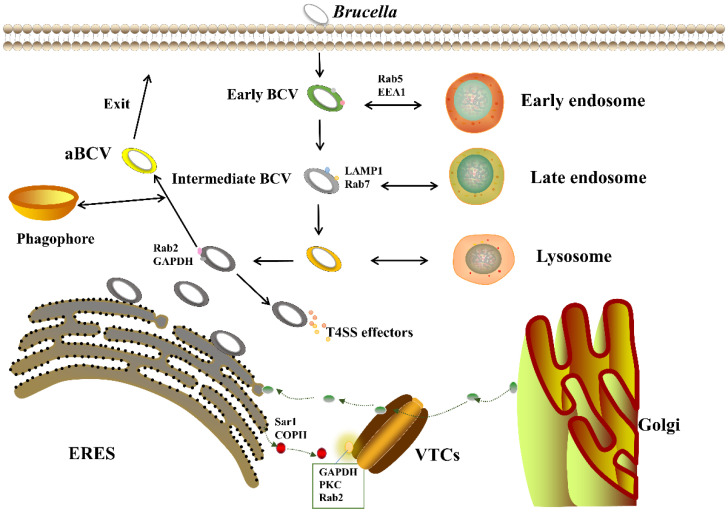
The intracellular lifecycle of *Brucella*. *Brucella* enters the host cell through a lipid raft and resides in phagocytic vesicles 8–12 h after infection. At the same time, forming acidified eBCVs through interactions with early and late endosomes and lysosomes, the host GTPase Rab7 participates in the maturation process of eBCVs, providing a physicochemical signal for T4SS expression. eBCV interacts with ERES to acquire endoplasmic reticulum and Golgi-derived membranes, leading to the biogenesis of rBCV. The host proteins Sar1, ire1α, Atg9, WIPI1 and the COG complex are involved in the generation of rBCVs. For 12–48 h, bacteria replicate extensively in rBCVs; after that, the rBCVs are captured into autophagosome-like structures and become autophagic BCVs (aBCVs); aBCV formation requires the host autophagy proteins Beclin1, ULK1 and Atg14. aBCVs have autophagosomal characteristics and are required for bacterial entry into and exit from cells.

*Brucella* can invade the body through skin mucosa, digestive tract and respiratory tract; then, it is enclosed in a membrane to form a *Brucella*-containing vacuole (BCV) [54]; subsequently, early endosomal markers (EEA-1, Rab5) and late endosomal markers (LAMP-1, Rab7, CD63, RILP) are obtained [55,56,57], which decreases PH and induces expression of VirB T4SS. Immediately after, *Brucella* interacts with endoplasmic reticulum exit sites (ERES) and transforms into the endoplasmic reticulum (ER)-derived replicative *Brucella*-containing vacuole (rBCV) with an endoplasmic reticulum structure and function [54]. After replication in the endoplasmic reticulum, rBCV becomes an autophagy-related *Brucella*-containing vacuole (aBCV); aBCV maturation completes the intracellular cycle of *Brucella* and facilitates subsequent cell-to-cell infection [58] (Figure 2).

### 4.1. VirB T4SS Action Stage

Various VirB system mutants are nontoxic in mouse experiments, suggesting that a functional VirB system is required for persistent *Brucella* infection in host cells [59]. Celli et al. [57] constructed VirB10 mutants to invade mouse BMDM cells; the survival rate of the VirB10 mutants was similar to that of wild-type S2308 during the first 4 h of infection, showing that the VirB system is not essential for the short-term survival of *Brucella*. However, 4 h after infection, VirB mutants persistently fuse with lysosomes and do not effectively control the maturation of bacterial vacuoles [60], whereas the wild type, upon interfusion with lysosomes, is able to lose the lysosomal component LAMP-1 through gaining the partner calnexin by capturing vesicles from ERES, and mediating the maturation of bacterial vacuoles (i.e., the biogenesis of rBCV) [27]. Persistent fusion of VirB mutants with lysosomes leads to bacterial killing, but interaction of VirB T4SS-released effectors with ERES to establish a replication niche can prevent this process [60,61,62].

ERES is a discrete region formed by the coated complex COPII on the endoplasmic reticulum [63,64], VirB acting on ERES is consistent with that Celli et al. [54] have shown eBCVs are converted to rBCVs via interaction with COPII-positive compartments mediated by VirB [54,55]. After rBCV formation, as *Brucella* proliferates, rBCVs are phagocytosed into autophagosome-like structures to become aBCVs [58]. Smith et al. [65] showed that aBCV formation and subsequent bacterial excretion are also dependent on VirB T4SS [65]. As the formation of aBCVs requires the involvement of host autophagy mechanisms [59], it is possible that specific VirB T4SS effectors regulate the related pathways to promote aBCV formation, but this result is not widely accepted at present. Taken together, we can speculate that the VirB system plays an essential role in the biogenesis of rBCV; in addition, it may also be involved in the formation of aBCV [66].

### 4.2. Cellular Pathways Affected by VirB T4SS

**Figure 3:**
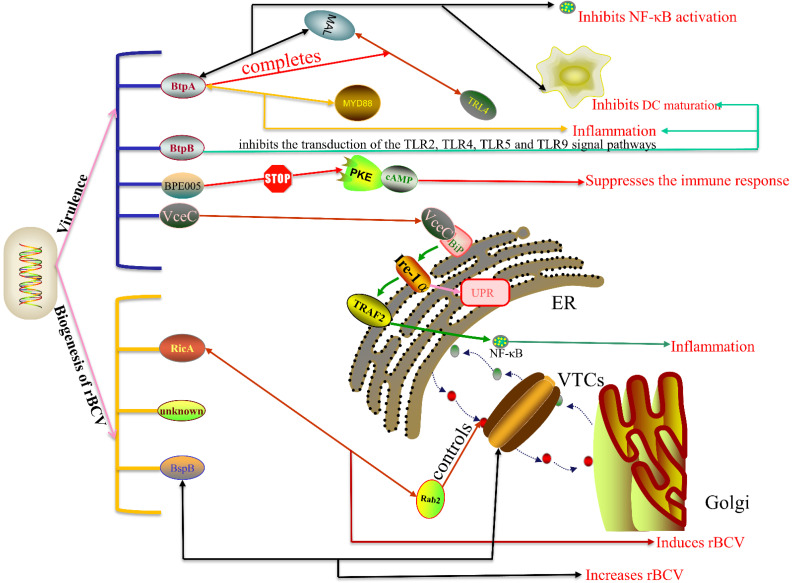
VirB T4SS affects cellular pathways. The role of T4SS effectors can be divided into two aspects: one is the influence through virulence, and the other is the influence on the formation of rBCV. In terms of virulence, the effector BtpA interacts with MAL and competes with it for TRL4, thus inhibiting the activation of NF-κB and the maturation of DC; effector BtpB inhibits DC maturation and induces inflammation by inhibiting transduction of the TLR2, TLR4, TLR5 and TLR9 signal pathways; effector BPE005 suppresses the immune response by inhabiting the combination of PKE and cAMP; effector VceC causes inflammation by activating UPR. In the biogenesis of rBCV, effector RicA reduces the Rab2 content in eBCV by interacting with it, thus reducing the formation of rBCV; effector BspB targets VTCs and interacts with COG to promote the formation of RBCV.

VirB T4SS secretes effectors into the host cell and alters specific cellular pathways, thus contributing to the virulence and the biogenesis of rBVC (Figure 3).

In pathogenetic terms, the T4SS effector VecC induces host inflammatory response via the unfolded protein response (UPR). VceC targets the ER and binds to Bip, leading to activation of the UPR. In mammalian cells, the UPR is activated by three different sensors on the endoplasmic reticulum (ER): inositol-requiring enzyme 1 (IRE1), protein kinase RNA (PKR)-like ER kinase (PERK) and activating transcription factor 6 (ATF6) [41]. Yuki et al.’s [41] experiments showed that during *Brucella* infection, the UPR can only be activated through the IRE1 pathway in a time-dependent manner [41]. Once activated, IRE1α transfers TNF receptor-associated factor 2 (TRAF2) to the ER and induces endoplasmic reticulum stress via the NF-κB pathway [67,68]. Then, endoplasmic reticulum stress through the NOD1/2-dependent TUDCA/KIRA6-sensitive pathway leads to inflammation [68]. When the ER is persistently or excessively stressed beyond the UPR limit, apoptosis is induced. The C/EBP-homologous protein (CHOP) is the most important UPR-mediated apoptotic pathway, VceC can interact with GRP78 to inhibit CHOP-induced apoptosis and promote persistent *Brucella* infection in cells [42].

The other two T4SS effectors related to inflammation are BtpA and BtpB; both of them contain the TIR domain. The TIR domain is a vital molecular component of TLR-mediated innate immune response. BtpA bears resemblance to the TIR domain adapter protein MAL and simulates the MAL function by combining with the plasma membrane using its N-terminal domain, thus competing with MAL for binding to TLR4 [46,47]. It can also interact with MAL through the Box1 region to decrease MAL phosphorylation levels, thereby inhibiting TLR4- and TLR2-mediated NF-κB activation and DC maturation [45,46]. BtpA interacts not only with MAL, but also with the death domain (DD) of MyD88, and this interaction is stronger than the interaction of BtpA with MAL [49]. As MyD88 serves as the central hub in inflammatory responses, BtpA may cause inflammation through it. Another T4SS effector, BtpB, has similar functions to BtpA, working with BtpA to control DC maturation and host inflammatory responses by inhibiting transduction of TLR2, TLR4, TLR5 and TLR9 signal pathways in vitro [48]. In conclusion, BtpA and BtpB can both induce inflammation and control host immunity.

In addition to BtpA and BtpB, T4SS effector BEP005 also controls host immune response by interfering with the cAMP/PKE signal pathway. The cAMP/PKE signaling pathway is thought to be involved in activation and proliferation of rat hematopoietic stem cells [69]. cAMP is a messenger for many hormones and neuropeptides, and the function of cAMP in eukaryotic cells is activated by PKE [70]. When hepatic stellate cells are infected, the cyclic nucleotide monophosphate-binding domain (cNMP) of the effector BPE005 blocks the binding of PKE and cAMP, thus inhibiting the secretion of MMP-9, inducing collagen deposition and TGF-β1 secretion. All that leads to partial suppression of the immune response, thereby establishing chronic *Brucella* infection [69].

In the aspect of rBVC biogenesis, T4SS effectors like BspB and RicA play a vital role. The biogenesis of rBVC needs functions of the host’s early secretory pathway, including Rab2, Rab1, Arf1 and host small GTPases Sar1. The eukaryotic protein component, Rab2, controls membrane transport between the ER and the Golgi, the T4SS effector RicA can interact with it directly or indirectly to control Rab2 replenishment on eBCV membranes, thereby reducing rBCV biogenesis and *Brucella* replication [38,71]. However, the T4SS effector BspB compensates RicA’s harmful activity on rBCV biogenesis and coregulates Golgi-derived vesicle transport in a compensatory manner [71]. BspB targets the Golgi and the ERGIC, wherein it interacts with the Golgi apparatus-associated conserved oligomeric Golgi (COG) complex, a regulator of vesicular traffic, and recruits COG-containing vesicles to the eBCV membrane, promoting the biogenesis of rBCV and bacterial replication [51,72].

Besides being regulated by the T4SS effectors identified above, the biogenesis of rBCV may also be affected by other unclear effectors. During infection, T4SS may secrete an effector to trigger the activation of IRE1, and IRE1 forms higher-order complexes with the assistance of host factor Yip1A, thus triggering the biogenesis of ER-derived autophagic vacuoles [41,60]. These vacuoles then fuse with endolysosomal vesicles; *Brucella* possibly intercept the vacuolar formation and obtain an ER-derived membrane [41,60,73]. Another hypothesis is that T4SS effectors activate IRE1 alpha-associated kinases (ASK1 and JNK1) and subvert a new host α-ulk1 signal axis. Kinases drive downstream proteins (ULK1, Atg9a, WIPI1 and Beclin1) assembly, which leads to the remodeling of the eBCV membrane and enables *Brucella* to establish a replication niche in cells [74]. It should also be noted that there is a eukaryotic protein component glyceraldehyde-3-phosphate dehydrogenase (GAPDH) on the rBCV membrane. GAPDH is normally located on VTCs between the ER and the Golgi apparatus, forms active complexes with Rab2, protein kinase C (PKCι/λ), and then constitutes a secretory pathway with COPI; the secretory pathway GAPDH/COPI/Rab2/pkcι/λ is essential for *Brucella* replication; inhibition of GAPDH leads to a reduction in *Brucella* replication [66,75]. This suggests that T4SS effectors may also affect the biogenesis of rBCV through this secretory pathway.

### 4.3. Regulatory Factors of VirB T4SS

The expression of the VirB system peaked during rBCV biogenesis, but the bacteria gradually began to decrease when replicating in the host cell, which indicates that the expression of the VirB system is strictly regulated in the host cell [76]. We divide the regulatory factors of the VirB system into direct regulatory factors and indirect regulatory factors.

Factors that directly regulate the VirB system include HutC IFH, quorum-sensing regulatory protein BlxR and autoinducers in the quorum-sensing system. The transcriptional repressor of the histidine utilization (Hut) genes HutC as a transcription factor directly controls the activity of the VirB promoter (P*_VirB_*). This regulation is related to the induction of the histidine utilization pathway. It may be a method to escape host immunity by sensing self-metabolism and developing adaptive response [65]. The other regulatory factor, the host integration factor IHF, competes with HutC for the binding of P*_virB_*. IHF is a protein known to be involved in transcriptional regulation of multiple bacterial genes. It specifically interacts with P*_virB_* and induces DNA bending with a bending angle of 50.36°, playing a vital role in the expression of VirB T4SS [65,77]. Quorum-sensing system is also directly involved in the expression of VirB T4SS. Autologous inducer is the first quorum-sensing regulator found in *Brucella*, contains N-dodecyl-DL-homoserine lactone (C12-HSL); can change the conformation of the quorum-sensing regulatory protein VjbR by binding to it [78]. One of the functions of VjbR is to confine individual bacteria to phagosomes during the early stages of intracellular infection [79]. Cell infection and cell death experiments have shown that the absence of VjbR in *Brucella* rough mutants downregulates VirB expression, thereby completely eliminating its toxicity in macrophages [80]. The combination of an autologous inducer and VjbR makes VjbR no longer act as a transcription activator or bind to P*_virB_*, thus inhibiting the expression of the VirB operon [78]. Another quorum-sensing regulatory protein BlxR negatively affects genes related to energy production and transformation, translation and protein modification, ribosome structure and biogenesis, carbohydrate and amino acid metabolism, directly binds to P*_virB_* and represses transcription of the VirB operon [81].

The other two factors indirectly regulating VirB T4SS are UDP-glucose pyrophosphorylase (UGPase) and Hfq. UGPase is a pyrophosphatase; can regulate the expression of virB proteins (virB3, virB4, virB5, virB6, virB8, virB9, virB10 and virB11) and T4SS effectors (vceC, btpA, btpB, ricA, bspB, bspC and bspF) by promoting the expression of topA, rpsL and BMEI1825 [82]. Another indirect regulator Hfq, a bacterial protein mediating RNA–RNA interactions, is induced at the later stage of infection and affects VirB1 protein production, probably by mediating the sRNA–mRNA interaction [83,84].

## 5. Conclusions Remarks

The intracellular replication of *Brucella* relies on the formation of T4SS encoded by VirB, which regulates host cell pathways through the release of effectors. VirB T4SS modifies the original vacuole into a replicative vacuole associated with the ER, ensuring bacterial survival, proliferation and egress. The VirB system plays a crucial role in *Brucella* intracellular infection, but the process of T4SS formation encoded by VirB protein interaction and the mechanism of T4SS effectors remain to be further investigated. It is of great significance not only for understanding the pathogenic mechanism of bacteria, but also for the immune response of eukaryotes, and will contribute to the development of clinical vaccines.

## Figures and Tables

**Table 1:** Necessity of proteins VirB1–12 in the VirB system for intracellular parasitism of *Brucella*.

VirB Protein	Species	Survival in Macrophages	Survival in the Mouse Model
VirB1	*Brucella abortus*	Necessary [12]	Dispensable [12]
VirB2	*Brucella abortus*	Necessary [12]	Necessary [12]
VirB3–6	*Brucella abortus*	Necessary [14]	Necessary [14]
VirB7	*Brucella abortus*	Necessary [14]	Dispensable [14]
VirB8–11	*Brucella abortus*	Necessary [14]	Necessary [14]
VirB12	*B. melitensis*, *B. abortus and B. suis*	Dispensable [13]	Dispensable [13]

## Data Availability

Not applicable.
